# Isolation, identification, and environmental adaptability of heavy-metal-resistant bacteria from ramie rhizosphere soil around mine refinery

**DOI:** 10.1007/s13205-017-0603-2

**Published:** 2017-04-08

**Authors:** Jie Jiang, Chaohu Pan, Aiping Xiao, Xiai Yang, Guimin Zhang

**Affiliations:** 1 College of Life Sciences, Hubei University, Wuhan, 430062 Hubei China; 2 Institute of Bast Fiber Crops, Chinese Academy of Agricultural Sciences, Changsha, 410205 Hunan China

**Keywords:** Ramie, Rhizosphere soil, Heavy-metal-resistant, Bacteria, Environmental adaptability

## Abstract

Six bacteria strains from heavy-metal-polluted ramie rhizosphere soil were isolated through Cd^2+^ stress, which were numbered as JJ1, JJ2, JJ10, JJ11, JJ15, and JJ18. Sequence alignment and phylogenic analysis showed that strain JJ1 belonged to *Pseudomonas*, strain JJ2 belonged to *Cupriavidus*, strains JJ11 and JJ15 belonged to *Bacillus*, and strains JJ10 and JJ18 belonged to *Acinetobacter*. The tolerance capability of all the strains was the trend of Pb^2+^ > Zn^2+^ > Cu^2+^ > Cd^2+^, the maximum tolerance concentration to Cd^2+^ was 200 mg/L, to Pb^2+^ was 1600 mg/L, to Zn^2+^ was 600 mg/L, and to Cu^2+^ was 265 mg/L. Strains JJ1, JJ11, JJ15, and JJ18 could grow well under pH 9.0, and strains JJ2, JJ11, and JJ18 could grow well under 7% of NaCl. The results showed that as a whole these strains had high environmental adaptability. This is the first report that heavy-metal-tolerant bacteria were found from ramie rhizosphere soil, which could be as a foundation to discover the relationship between ramie, rhizosphere bacteria and heavy metals.

## Introduction

Over exploitation for mineral resources has overloaded the endurance capability of the environment, the pollution caused by the release of heavy metals during mining is getting more and more serious (Cheng 2003). The heavy metals accumulated in soil are absorbed and accumulated in plants, and then transferred into human body through food chains. Which is seriously harmful for human health, some cancers and diseases are hence induced (Cuningham et al. 1995). The technologies applied with physical and chemical methods to restore polluted soils have been carried on since 1980s (Ellis et al. 2003). These technologies, to a certain extent, can restore the polluted soil, but the processes are complicated and secondary pollution might be caused (Chunfa et al. 2014; Wenqing et al. 2014).

Compared with physical and chemical methods, bio-restoration technology is gotten attention considerably because of its advantages of low cost, little harmfulness for environment, and high efficiency (Hong et al. 2013). The technology with the combination application of plant-microorganism is the most hot research topic on restoring the heavy-metal-polluted soils (Glick 2003; Hooda 2007).

Most heavy metals are toxic for cell growth, such as Cd, Hg, and Pb, but some microorganisms can endure, resist, and absorb heavy metals by changing valence state, metabolic pathway, producing secondary metabolites, etc. (Braud et al. 2006; Yingli et al. 2013). Large quantity of microorganisms which can resist heavy metals are isolated from soil, most of them are bacteria. The main heavy-metal-resistant bacteria existing in soil are *Acinetobacter*, *Achromobacter*, *Azospirillum*, *Alcaligenes*, *Pseudomonas*, *Psychrobacter*, *Bacillus*, etc. (Gray and Smith 2005; Ying et al. 2013).

As an important fiber crop growing widely in south of China, ramie (*Boehmeria nivea* (L.) Gaudich.) is a perennial plant with strong roots, which is easy to form a stationary rhizosphere environment. This crop can grow well in heavy-metal-polluted soils such as mining areas, heavy metals are accumulated mainly in its roots, stems, and leaves (Jianping et al. 2003; Ying et al. 2005). When stressed with the mixture of Cd, Sb, and Pb, the absorption tendency in ramie plants is Cd > Sb > Pb, the highest accumulation aboveground parts are up to 335.74, 157.55, and 92.31 mg/kg respectively, which shows that ramie has good accumulation and transference capability for Cd/Sb/Pb (Guiyuan et al. 2012). The average accumulations of Cd, Pb, As, Sb, Zn, and Cu are up to 0.11, 1.17, 0.72, 7.97, 6.71, and 1.69 kg/hm^2^ respectively in ramie planted experimentally in three mine areas of Shimen, Lengshuijiang, and Liuyang in Hunan Province of China (Wei et al. 2011). Ramie is not food crop with strong heavy-metal-tolerance and environmental adaptability, compared with food crops and other plants with low mass yields, it is a much preferable plant to restore heavy-metal-polluted soils.

In this paper, some heavy-metal-resistant bacteria were isolated and identified from ramie rhizospheric soil around mine refinery of Zhuzhou Smelter Group Co., Ltd. in Hunan province of China. The tolerance to heavy metals, the adaptability of pH, and salinity were investigated so as to lay a foundation for discovering the relationship metabolism between ramie, rhizosphere bacteria, and heavy metals, and also for restoring the polluted soils by applying the combination function of ramie and rhizosphere bacteria.

## Materials and methods

### Soil sample origin

The rhizospheric soil samples were shaken off from the roots of ramie growing around Zhuzhou Smelter Group Co., Ltd. in Hunan province of China. The ramie plants were growing in the soils covered with mine dust about 10–15 cm of thickness. The soils were collected in sterile bags packed in a portable ice box with −4 °C, and then delivered to the laboratory within 1 h.

## Methods

### Isolation of Cd^2+^-resistant bacteria

3 g of soil sample was aseptically put into 100 mL sterilized water, 28 °C, 150 r/min for 30 min in shaker. 1 mL solution was aseptically and gradually diluted in 9 mL sterile water. 0.1 mL of the dilution series of 10^−3^, 10^−4^, 10^−5^, and 10^−6^ was separately drawn and spread on LB solid medium containing yeast extract 5 g/L, peptone 10 g/L, NaCl 10 g/L, agar 15 g/L, and Cd^2+^ 100 mg/L. The plates were placed upside down at 28 °C in incubator till the colonies grew up. Single colonies were picked out and isolated through streak plate cultivation to get pure culture. The purified and grown well colonies were regarded as the Cd^2+^-resistant bacteria and stored under −80 °C.

### 16S rDNA sequence analysis and phylogenetic tree construction

Single Cd^2+^-resistant colonies were inoculated in LB liquid mediums in shaker, 28 °C, 150 r/min culturing overnight. Silica gel crushing method was used to extract and separate chromosome DNA of the strains (Stackebrandt and Goodfellow 1991). The pairs of universal primers were used to amplify, the forward primer was 27F: 5′-AGAGTTTGATCCTGGCTCAG-3′, the reverse primer was 1492R: 5′-ACGGCTACCTTGTTACGACTT-3′ (Masatoshi and Sudhir 2000). The mixtures used for PCR amplification contained extracted DNA 0.5 ng, forward primers 2.5 μL, reverse primers 2.5 μL, dNTP 5 μL, Taq enzyme buffer 5.0 μL, and Taq enzyme 0.5 μL, and highly purified water was added up to 50 μL. The temperature program for amplification was as follows: pre-denaturation at 94 °C for 5 min, denaturation at 94 °C for 35 s, anneal at 55 °C for 1 min, extension at 72 °C for 1 min 30 s, total 30 recycles, final extension at 72 °C for 4 min. 150 μL PCR products were electrophoresed with 1.0% agarose gel at 90 V for 90 min. The bands of 15 kb DNA were cut down quickly under ultraviolet light after being stained with ethidium bromide, and put in 1.5 mL sterilized Eppendorf. The subsequent purification was processed according to DNA extraction kit (Axygen Co., Ltd.).

The purified products were joined with vector pMD18-T, transplanted into *E. coli* Top10 competent cells to screen recombinants. The universal primers M13 were used to amplify the recombinants, the reaction mixtures and conditions of PCR were the same as the above mentioned. The positive clones were about 1500 bp, and the false positive clones were about 150 bp. The targeted recombinants were sequenced and identified by GenScript Co., Ltd. (Nanjing). The sequence results were uploaded into Genbank and compared with the reported sequences by BLAST alignment in NCBI. The sequences with high homology were selected to construct phylogenetic tree based on Neighbor-Joining Mega 6.0. The bootstrap was designed as 1000 times (Tamura et al. 2013).

### Tolerance concentrations to heavy metals

The initial solutions of 1.0 g/L CdCl_2_, 2.0 g/L Pb(NO_3_)_2_, 2.0 g/L ZnSO_4_, and 2.0 g/L CuSO_4_ were sterilized at 121 °C for 15 min, and aseptically added into the sterilized LB solid mediums to prepare the selective mediums containing 100, 150, 200, 250, 300 mg/L Cd^2+^, 1200, 1400, 1500, 1600, 1800 mg/L Pb^2+^, 130, 150, 185, 265, 300 mg/L Cu^2+^ and 200, 300, 400, 500, 600 mg/L Zn^2+^, respectively. The isolated strains were lined to inoculate in these selective mediums containing series of concentrations of heavy metals, culturing in incubator at 28 °C for 1–3 days. The healthy and well grown colonies were regarded as bacteria capable of resisting to the heavy metals.

### Adaptability to pH and salinity

The pHs of LB liquid mediums were adjusted to pH 4.0, pH 5.5, pH 6.0, pH 7.0, and pH 9.0 with the solutions of 2 mol/L HCl and 2 mol/L NaOH. The activated bacteria were inoculated in the liquid mediums with the dose of 2% respectively, culturing under 28 °C, 250 r/min for 18 h in shaker. The OD values of bacteria suspensions with different pHs were determined with spectrophotometer under 600 nm (OD_600_). The same dosage of bacteria was inoculated in the LB liquid mediums containing 1, 2, 3, 5, and 7% of NaCl , culturing under 28 °C, 250 r/min for 18 h in shaker. The OD values of bacteria suspensions with different salinities were determined with spectrophotometer under 600 nm (OD_600_).

## Results

Six bacteria strains with the capability of resistance to Cd^2+^ were isolated and numbered as JJ1, JJ2, JJ10, JJ11, JJ15, and JJ18. The maximum tolerances for the six strain to Cd^2+^, Pb^2+^, Cu^2+^ and Zn^2+^ were 200 mg/L, 1600 mg/L, 265 mg/L and 600 mg/L shown as Table 2.

The sequence results were uploaded into Genbank, the accession number was KP226587–KP226594. The 16S rRNA gene sequences of the six strains were shown as Table 1. The maximum similarity was up to 99% compared with the reported model bacterium for all the six strains. Among them, strain JJ11 had the similarity of 99% with the model strain *Bacillus mycoides* IARI-JR-40 (KF054993.1). Strain JJ15 had the similarity of 99% with the model strain *Bacillus salmalaya* 139SI (KM051837.1). The 16S rRNA gene sequences of strains JJ1, JJ2, JJ10, and JJ18 had the maximum similarity with the strains of *Chryseobacterium*, *Cupriavidus, Pseudomonas* and *Acinetobacter,* respectively (Table 2).

**Table 1:** Comparison results of 16 S rRNA gene sequences of the 6 bacteria with the reported ones in Genbank

Experimental strains (accession numbers)	Reported strains with maximum similarity (accession numbers)	Identity (%)
JJ1 (KP226587)	*Chryseobacterium indoltheticum* LMG 4025 (NR042926.1)	99
JJ2 (KP226588)	*Cupriavidus oxalaticus* NBRC 13593 (R113619.1)	99
JJ10 (KP226592)	*Pseudomonas helmanticensis* OHA11T (HG940537)	99
JJ11 (KP226591)	*Bacillus mycoides* IARIJR 40 (KF054993.1)	99
JJ15 (KP226593)	*Bacilluss almalaya* 139SI (KM051837.1)	99
JJ18 (KP226594)	*Acinetobacter tjernbergiae* Z2STSA 11 (KC213887.1)	99

**Table 2:** Maximum tolerance concentrations of the 6 bacterial strains to 4 heavy metals

Serial no.	Maximum concentration (mg/L)			
	Cd^2+^	Pb^2+^	Cu^2+^	Zn^2+^
JJ1 (KP226587)	200	1500	265	600
JJ2 (KP226588)	100	1600	185	500
JJ10 (KP226592)	100	1400	>265	500
JJ11 (KP226591)	100	1600	150	500
JJ15 (KP226593)	100	1600	265	500
JJ18 (KP226594)	200	1400	130	200

**Fig. 1:**
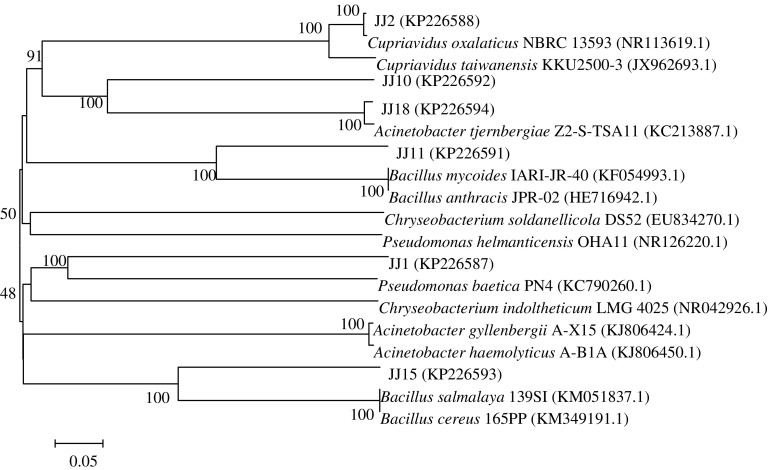
Phylogenetic tree based on 16 S rRNA gene sequences of 6 Cd^2+^-resistant bacteria with Neighbor-Joining method. The *numbers on the branches* indicate the bootstrap values over 48%, accession numbers in GenBank are given in *parentheses*

The phylogenetic tree was shown as Fig. 1 based on 16S rRNA gene sequences. With the bootstrap value of 100%, strain JJ2 was grouped with *Cupriavidus oxalaticus* NBRC 13593 (NR113619.1) and *Cupriavidus taiwanensis* KKU 2500-3 (JX962693.1), and had a closer genetic relationship with *C. oxalaticus*. Thus, strains JJ2 belonged to *Cupriavidus*. Strain JJ11 was grouped with *Bacillus mycoides* IARI-JR-40 (KF054993.1) and *Bacillus anthracis* JPR-02 (HE716942.1), and had a closer genetic relationship with *B. mycoides*. Strain JJ15 was grouped with *Bacillus salmalaya* 139SI (KM051837.1) and *Bacillus cereus* 165PP (KM349191.1), and had a closer genetic relationship with *B. salmalaya.* Thus, strains JJ11 and JJ15 belonged to *Bacillus*. Strain JJ1 had the maximum similarity with *Chryseobacterium indoltheticum* LMG 4025 (NR-042926.1), but was grouped and had a closer genetic relationship with *Pseudomonas baetica* PN4 (KC790260.1). Thus, strain JJ1 might belong to *Pseudomonas*. Strains JJ18 was grouped with *Acinetobacter tjernbergiae* Z2-S-TSA11 (KC213887.1). Strain JJ10 was grouped with strain 18 because of the closest distance showing on the phylogenetic tree, although strain JJ10 had the similarity of 99% with *Pseudomonas helmanticensis* OHA11T (HG940537). Thus, strains JJ10 and JJ18 might belong to *Acinetobacter*.

The whole trend of tolerance capability to heavy metals showed as Pb^2+^ > Zn^2+^ > Cu^2+^ > Cd^2+^, the maximum tolerance concentration to Pb^2+^ was up to 1600 mg/L, to Zn^2+^ was up to 600 mg/L, to Cu^2+^ was up to 265 mg/L, and to Cd^2+^ was up to 200 mg/L. Relatively speaking, strains JJ1, JJ10, and JJ15 had stronger tolerance capability to heavy metals than the others.

**Fig. 2:**
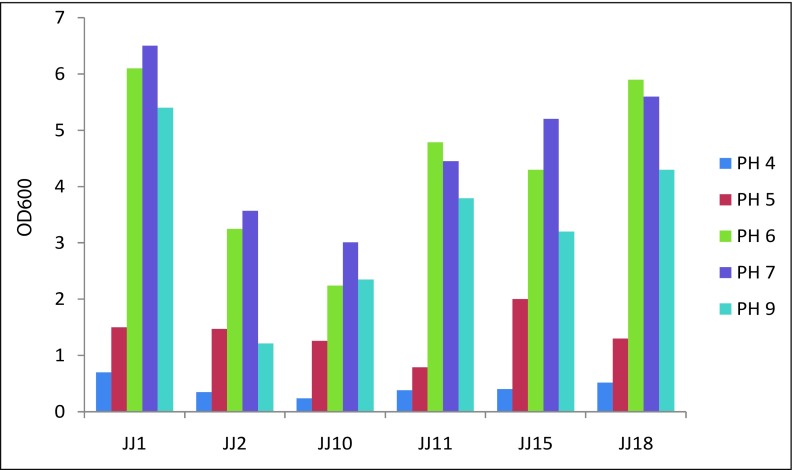
Growth diagram of the 6 strains from pH 4.0 to pH 9.0

The growth diagram of the six bacteria under different pHs was shown as Fig. 2. All the strains could grow under pH 4.0–9.0, but there was much difference under different pHs for the six bacteria. The range of pH 6.0–7.0 was optimal, and the growth rate decreased rapidly with the decrease of pH for all the six strains. Although the growth rate decreased slowly with the increase of pH, the strains grew better than under low pH. Therefore, the six bacteria had relative strong adaptability under neutral and alkali conditions, especially for JJ1, JJ11, JJ15, and JJ18.

**Fig. 3:**
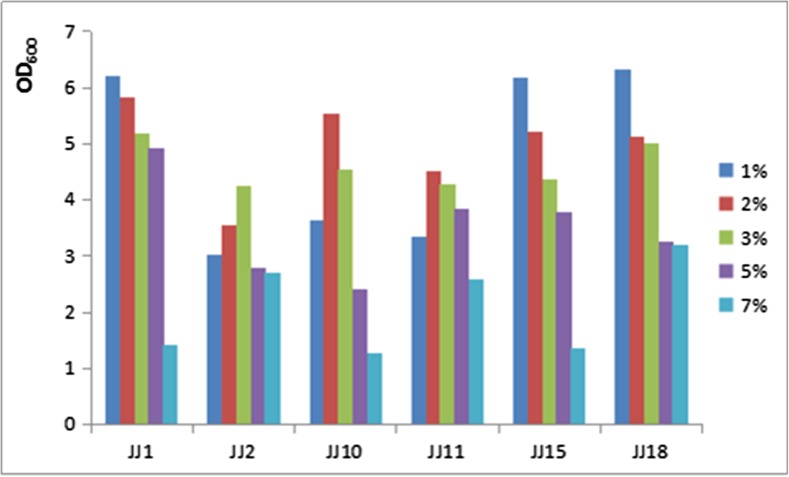
Growth diagram of the 6 strains from 1 to 7% of NaCl

The growth diagram of the six strains was shown as Fig. 3 under different salinities. All the six strains could grow at the range of 1–7% NaCl, there was no evident inhibition under high NaCl concentration. The growth rate decreased with the increase of the concentration of NaCl for all the six strains as a whole. 1% NaCl was optimal for JJ1, JJ15, and JJ18, 2% NaCl was optimal for JJ10 and JJ11, and 3% was optimal for JJ2. The growth rate for strains JJ2, JJ10, and JJ11 showed the trend from slow to quick, then to slow with the increase of NaCl concentration. Strains JJ1, JJ11, JJ15, and JJ18 could grow well under 5% NaCl, while strains JJ2, JJ11, and JJ18 could still grew well under 7% NaCl.

## Discussion

Zhuzhou city in Hunan province is a heavy industry city, the soil near some mine refineries are polluted heavily by heavy metals such as Cu, Pb, Zn, and Cd, especially the pollution degree of Cd has been in a severity (Chaozhuang et al. 2008). Six bacterial strains were isolated and identified from the rhizosphere soil of ramie growing around Zhuzhou Smelter Group Co., Ltd. in Hunan province of China. Being compared with the reported 16S rRNA gene sequences in Genbank, three of them belonged to *Proteobacteria*, two of them belonged to *Firmicutes*, and one of them belonged to *Flavobacteria*. Strain JJ2 had the same genus with *Cupriavidus metallidurans* which is the type strain of anti-heavy metal bacteria in soil (Lazzaro et al. 2008). Strains JJ10, J11, JJ15, and JJ18 had the same genus with the reported heavy-metal-resistant bacteria. The similarity between strain JJ11, strain JJ15, and *Bacillus spp*., was 99%, and these reported kinds of bacteria are very rich in the heavy-metal-polluted soils (Ellis et al. 2003; Leni et al. 2010), this experiment results also testified the viewpoint. The maximum tolerance concentration to single heavy metal for *E. coli* was as the tendency of Cd^2+^ 56 mg/L, Pb^2+^ 41.4 mg/L, Cu^2+^ 64 mg/L, and Zn^2+^ 65 mg/L (Nies 1999). The resistance capability of the six strains to the four heavy metals was much stronger than *E. coli*. The highest tolerance to Cd^2+^ for strain No.9 originated from radiaiton-polluted soil was up to 2100 mg/L (Jing et al. 2013). Two strains of *Pseudomonas aeruginosa* and *Enterobacter cloacae* isolated from Cd-polluted soil had the tolerance of 400 and 300 mg/L to Cd^2+^ respectively (Xiaoyan et al. 2015). Compared with the reported heavy-metal-resistant bacteria from other environment, the six strains might not have the highest tolerance to one single heavy metal, but they showed strong resistant capability to several heavy metals at the same time (Yuanyuan et al. 2005).

Microorganisms which have the optimal pH 8.0, or between pH 9.0–10.0, are categorized into alkaliphiles. Microorganisms which have no optimal alkali pH but can grow well under alkali condition are categorized into alkalitolerants (Yanhe 1999). The six bacterial strains could grow under pH 4.0–9.0; the optimal pH was pH 6.0–7.0. Strains JJ1, JJ11, JJ15, and JJ 18 grew normally under pH 9.0, which demonstrated that the four strains had strong alkali adaptability and could be regarded as alkolintolerant bacteria. Based on the definitions, the microorganisms which can grow under different salinities are categorized as halotolerant and halophiles. Among them, microorganisms which can grow well under the salinity of 2.93–14.63% are categorized into moderately halophilic bacteria and microorganisms which can grow well under the salinity of 14.63–30.4% are categorized into extremely-halophilic bacteria (Kushner 1978; Galinski and Trüper 1994). The six bacterial strains grew under 1–7% of NaCl. There was no evident inhibition under high concentration of NaCl, especially for strains JJ2, JJ11, and JJ18. Therefore, the three strains could be regarded as moderately halophilic bacteria. There are few reports on alkali-tolerant or halotolerant bacteria which could be tolerant to heavy metals at the same time (Aimin 2005), while the bacteria with comprehensive function is more valuable than those with single function.

To sum up, this paper describes that heavy-metal-tolerant bacteria are first isolated from ramie rhizosphere soil polluted heavily with heavy metals. These six strains had a wide range of adaptability under different environmental conditions. Based on the tolerance capability to heavy metals, pH and salinity, strains JJ1, JJ11, JJ15, and JJ18 could be candidate materials for restoring heavy-metal-polluted soils with alkalinity. Strains JJ2, JJ11, and JJ18 could be used as candidate materials for restoring heavy-metal-polluted soils with high salinity. The metabolism of resistance to heavy metals for these bacterial strains is needed to be discovered further. The obtained results may lay a foundation for discovering the relationship between rhizospheric soil, microorganism, and ramie plant.
